# Transgenerational Transmission of the *Glossina pallidipes* Hytrosavirus Depends on the Presence of a Functional Symbiome

**DOI:** 10.1371/journal.pone.0061150

**Published:** 2013-04-22

**Authors:** Drion G. Boucias, Henry M. Kariithi, Kostas Bourtzis, Daniela I. Schneider, Karen Kelley, Wolfgang J. Miller, Andrew G. Parker, Adly M. M. Abd-Alla

**Affiliations:** 1 Entomology and Nematology Department, University of Florida, Gainesville, Florida, United States of America; 2 Insect Pest Control Laboratory, Joint FAO/IAEA Programme of Nuclear Techniques in Food and Agriculture, Vienna, Austria; 3 Laboratory of Virology, Wageningen University, Wageningen, The Netherlands; 4 Laboratories of Genome Dynamics, Center of Anatomy and Cell Biology, Medical University of Vienna, Vienna, Austria; 5 ICBR Electron Microscope Facility, University of Florida, Gainesville, Florida, United States of America; Wuhan Bioengineering Institute, China

## Abstract

The vertically transmitted endosymbionts (*Sodalis glossinidius* and *Wigglesworthia glossinidia*) of the tsetse fly (Diptera: Glossinidae) are known to supplement dietary deficiencies and modulate the reproductive fitness and the defense system of the fly. Some tsetse fly species are also infected with the bacterium, *Wolbachia* and with the *Glossina* hytrosavirus (GpSGHV). Laboratory-bred *G. pallidipes* exhibit chronic asymptomatic and acute symptomatic GpSGHV infection, with the former being the most common in these colonies. However, under as yet undefined conditions, the asymptomatic state can convert to the symptomatic state, leading to detectable salivary gland hypertrophy (SGH^+^) syndrome. In this study, we investigated the interplay between the bacterial symbiome and GpSGHV during development of *G. pallidipes* by knocking down the symbionts with antibiotic. Intrahaemocoelic injection of GpSGHV led to high virus titre (10^9^ virus copies), but was not accompanied by either the onset of detectable SGH^+^, or release of detectable virus particles into the blood meals during feeding events. When the F_1_ generations of GpSGHV-challenged mothers were dissected within 24 h post-eclosion, SGH^+^ was observed to increase from 4.5% in the first larviposition cycle to >95% in the fourth cycle. Despite being sterile, these F_1_ SGH^+^ progeny mated readily. Removal of the tsetse symbiome, however, suppressed transgenerational transfer of the virus via milk secretions and blocked the ability of GpSGHV to infect salivary glands of the F_1_ progeny. Whereas GpSGHV infects and replicates in salivary glands of developing pupa, the virus is unable to induce SGH^+^ within fully differentiated adult salivary glands. The F_1_ SGH^+^ adults are responsible for the GpSGHV-induced colony collapse in tsetse factories. Our data suggest that GpSGHV has co-evolved with the tsetse symbiome and that the symbionts play key roles in the virus transmission from mother to progeny.

## Introduction

Tsetse flies (*Glossina* spp.), obligatory blood-feeders, are distributed throughout tropical sub-Saharan Africa and are vectors of *Trypanosoma* spp. that cause African animal trypanosomosis (AAT) or nagana in livestock and human African trypanosomosis (HAT) or sleeping sickness in humans [1], [2]. In many parts of sub-Saharan Africa, AAT and the presence of tsetse are considered as major obstacles to the development of more efficient and sustainable livestock production systems and represent one of the most important root causes of hunger and poverty [3], [4]. The most widely used method to manage AAT is through the prophylactic and curative treatment of livestock with trypanocidal drugs [5]. However, it is generally accepted that controlling the vector, the tsetse fly, remains the most efficient and sustainable way of managing AAT [1], [6]. The use of sterile insects as part of an area-wide integrated pest management (AW-IPM) [7], [8] approach is considered a very powerful control tactic for the sustainable eradication of tsetse flies as amply demonstrated on the island of Unguja, Zanzibar [9]. The efficient implementation of sterile insect technology (SIT) for tsetse control depends on the successful establishment of tsetse factories to produce high quality males capable of competing with wild males for mating with wild tsetse females [10].

Many of the tsetse species, including *Glossina pallidipes*, harbour a virus belonging to the family *Hytrosaviridae* [11]. The *G. pallidipes* salivary gland hypertrophy virus (GpSGHV), a rod-shaped dsDNA virus, replicates in the nucleus and acquires a fragile outer envelope in the cytoplasm [12], [13]. In tsetse flies, the infection can exhibit two phenotypes; a chronic non-debilitating asymptomatic (latent) infection and an acute, symptomatic infection that leads to reproductive dysfunction and colony collapse [14]. Incidence of asymptomatic infections can be high in both field and colonized tsetse fly species [15]. Asymptomatic infections are most likely maintained through vertical transmission either via the milk gland secretions or through gonadal tissues. The low virus titre in these asymptomatic flies does not cause measureable impacts on host fitness. However, under yet undefined conditions, the asymptomatic state is altered via a massive up-regulation of replication in the salivary gland cells that results in the detectable salivary gland hypertrophy (SGH^+^) syndrome. Symptomatic infections in tsetse flies directly impact various host fitness parameters [16], [17]. In natural populations, that harbour high levels of asymptomatic infections, the incidence of the SGH+ is low and ranges from 0–5% [15]. However, in mass-rearing facilities where adult flies are membrane-fed on a common blood source, the virus released into the blood via saliva from flies displaying SGH^+^ can be transmitted *per os* to asymptomatic flies thus boosting infection levels [16].

In addition to the hytrosavirus, tsetse fly species harbour a complex symbiome comprised of obligate and facultative symbiotic bacteria that are critical to their nutritional and reproductive fitness [18]–[22]. *Wigglesworthia glossinidia* (family Enterobacteriaceae), the obligate primary mutualist that is harboured in bacteriocytes localized in the anterior midgut, provides metabolic supplementation to this hematophagous insect, producing vitamins that are absent in blood meals [23], [24]. In addition, tsetse flies are known to harbour secondary symbionts such as the commensal *Sodalis glossinidius* (family Enterobacteriaceae) that is found both as extracellular and intracellular secondary symbiont in tsetse flies [19], [25]. It has been proposed that the metabolic machineries of *Wigglesworthia* and *Sodalis* complement each other in thiamine biosynthesis [26]. Both *Wigglesworthia* and *Sodalis* are maternally transmitted; in these adenotrophic viviparous females both bacteria have the ability to enter the milk glands, from which they are transmitted to developing larva [27]. Pregnant females provide nutrition amended with these symbionts to their offspring throughout the entire larval stage; a single fully developed larva is larviposited at 10–14 days intervals. A third bacterium, *Wolbachia pipientis*, detected in certain tsetse fly populations, is an intracellular alpha-proteobacterium that is transmitted from the mother to the egg cytoplasm. In insects, *Wolbachia* can affect life history traits, including the host reproduction, behaviour, immune competence, development and longevity [28], [29], and their removal by antibiotic treatment can impact fitness [30], [31].

This study was initiated in response to numerous reports on the role that bacterial symbionts play in maintaining the “immune status” in various insects with disruption of host symbiont homeostasis in these hosts resulting in increased susceptibility to disease [32]–[37]. Importantly, the bacterial symbionts associated with tsetse flies have also been implicated in modulating the immune status of these insects. In *G. morsitans morsitans*, the primary symbiont *Wigglesworthia* produces metabolites in the midgut bacteriome that down-regulate auto-activation of the innate defence systems that subsequently can impact fly fitness [38]. At another level, the maternal transfer of *Wigglesworthia* to the F_1_ progeny via milk gland secretions up-regulates the cell defence system; removal of symbionts with antibiotics results in the F_1_ being immuno-compromised [39].

Hence, we speculated that alterations in the symbiome (*Sodalis*, *Wigglesworthia*, and/or *Wolbachia*) would impact either the levels of GpSGHV or expression of SGH^+^ symptoms in adult *G. pallidipes*. Therefore, we set up a series of bioassays to examine the effects of antibiotic treatment on both asymptomatic flies and flies injected with infected gland extracts. Our working hypothesis was that the antibiotic-induced immune activation/suppression would alter virus titre in adults or in the F_1_ progeny and trigger changes in the expression of SGH^+^ symptoms. Using quantitative PCR (qPCR) we measured the impact of the treatments on the relative levels of the symbiome in adult *G. pallidipes*. In the light of the observed delayed effects of virus injections on tsetse flies, we followed all treatments through to the emergence of the F_1_ progeny.

## Materials and Methods

### Insect rearing and handling

All experiments were carried on a *G. pallidipes* colony originating from pupae collected in Tororo, Uganda in 1975, colonized initially at the University of Leiden, The Netherlands, and transferred subsequently to the Insect Pest Control Laboratory, Seibersdorf, Austria in 1982. Unless otherwise stated, all experimental flies in this study were fed on heated, defibrinated bovine blood (Svaman spol s.r.o., Myjava, 90701, Slovakia) for 10–15 minute intervals, three times per week, using a membrane-feeding technique [40]. Pupae produced from sequential larviposition events were collected and incubated at 24°C until emergence.

### Preparation of virus inoculum

In this study, the virus inoculum was prepared from male *G. pallidipes* exhibiting overt SGH^+^ symptoms selected from the colony [16], [41] For each inoculum, a pair of intact hypertrophied salivary glands was aseptically dissected out and gently homogenized in 1 ml of filter-sterilized PBS. The homogenate was briefly centrifuged (2,000× g; 5 min; 4°C) to remove tissue debris. The supernatant was collected and filtered through a 0.45 micron filter. Due to the fragile nature of the virus particles [13], the virus filtrate was kept on ice throughout and used within 1 h of preparation. Since there is no cell culture available for GpSGHV propagation, end-point titration assay to estimate infectious virus particles in the inoculum was not possible. Instead, the virus copy numbers present in the filtrate were estimated by quantitative PCR (qPCR) using a standard prepared by amplified GpSGHV ORF005 (*odv-e66* gene) amplicons as previously described [42]. By this method, ∼1×10^6^ virus copies were estimated to be present in a 2 µl aliquot of gland filtrate used as inoculum in the experiments described below.

### Kinetics of virus replication in adult *G. pallidipes*

To investigate GpSGHV replication in *G. pallidipes*, teneral (newly emerged, unfed) flies were pre-screened for GpSGHV infection by a non-destructive PCR as previously described with slight modification [14]. In brief, total DNA was extracted from one intermediate excised leg of a teneral fly using ZR DNA genomic kit (Zymo Research, California, USA) according to the supplier's instructions. The DNA was eluted in 25 µl elution buffer, and the PCR was carried out using the GpSGHV ORF005 primers previously described [14]. The PCR products were analysed on a 1% agarose gel. PCR-negative individuals were selected for virus-injections.

For injections, 6 groups of flies, each consisting of 4 males and 12 females, were injected with 2 µl of the virus suspension (diluted 10^−4^ in filter-sterilized phosphate buffered saline (PBS) and the same number of flies were mock-injected with PBS. Each treatment was replicated 3 times. Immediately after injection, flies (4 males and 12 females) from one group were frozen at −20°C for subsequent quantitation of virus copy numbers by qPCR analysis. The remaining 5 groups were maintained on normal blood meal for 1, 3, 5, 7 and 9 feeds. Forty-eight hours after the end of the respective number of feeds, the flies were frozen at −20°C for later qPCR analysis.

### Assays with virus and ampicillin treatments

Groups of 130 female and 30 male cold-anesthetized, newly emerged flies were injected into the thoracic cavity with either 2 µl of undiluted viral gland filtrate or 2 µl of filtered PBS prepared as described in the previous section. Virus- and PBS-injected flies were subsequently subdivided into cages, two containing virus-injected flies and two containing PBS-injected flies. One cage from each treatment (virus- or PBS-injected) was fed on normal blood; the second cage was fed blood supplemented with 40 µg/ml of the penicillin-based antibiotic ampicillin (Sigma) three times per week for the duration of the experiment. Based on prior research on *G. moristans moristans* [30] this antibiotic treatment is expected to have little or no impact progeny production by treated females but should generate *Wigglesworthia*-free F_1_ sterile flies. The entire experiment was replicated three times. In addition to these three replicates, two additional cages containing 60 females and 15 males were inoculated with the virus preparation. Five adults were removed weekly from each cage, sexed, dissected for determination of the incidence of SGH^+^, and frozen for subsequent DNA extraction. F_1_ pupae were collected, placed individually in dated cells and incubated at 24°C until adult emergence. Newly emerged F_1_ adults were sexed, dissected for incidence of SGH^+^ and frozen individually for subsequent DNA extraction. Virus copy numbers in the extracted DNA were determined by qPCR.

### Production and release of GpSGHV in salivary secretions

The ability of both asymptomatic and symptomatic infected flies to produce and release GpSGHV in salivary secretions was examined. Groups of 24 flies (6 males and 18 females) were either mock-injected with PBS- or virus-challenged as described above. The flies were kept in individual cells and subsequently fed on 250 µl of normal blood each for 21 days post infection (dpi). After the last blood meal, approximately 100 µl of the remaining blood was collected to estimate copies of virus particles released into the blood meals during the feeding event. DNA was extracted from both the collected blood and whole flies bodies using DNeasy Blood and Tissue Kit (QIAGEN, GmbF, D.40724, Hilden) according to manufactures instruction. The virus copy number released into the blood was estimated by qPCR [42].

### DNA extraction, end-point PCR and quantitative PCR

DNA was extracted from the entire fly from both the sampled parents and F_1_ adults using the DNeasy 96 DNA Blood Kit (Qiagen). For quality control of the DNA extraction step, samples were diluted in TE buffer, PCR amplified with primers designed for the tsetse housekeeping β-tubulin gene (**Table S1**), and visualized on ethidium bromide-stained 1% agarose gels. The few extractions that produced weak bands (or no product) were excluded from the subsequent analyses. Subsets of samples producing homogenous and strong positive amplicons with the housekeeping gene primers [43] were subsequently subjected to a series of qPCR reactions to determine the GpSGHV copy numbers [42] in both the parents and the emerging F_1_ adults, (**Table S1**).

Primers designed from the flagellin C (FliCF) gene of *S. glossinidius* isolated from *G. morsitans morsitans* were used to quantitate the *Sodalis* levels in the *G. pallipides* adults (**Table S1**). Initial attempts to use the primers designed from the thiamine biosynthesis protein *(thiC)* gene of *W. glossinidia* (40) failed to amplify product from the genomic *G. pallipides* DNA samples. Using degenerate primers designed from the open-reading frames of *thiC* genes (BA000021.3, CP003315.1) with the CODEHOP program [44]we were able to successfully amplify and sequence a partial (645 bp) *thiC* sequence of the *W. glossinidia* of *G. pallipides* (GenBank Acc KC470073). Primers designed from the conserved ThiC domain were used to quantitate the levels of *W. glossinidia* in the *G. pallipides* adults (**Table S1**). To prepare qPCR standard curves for both the viral and bacterial amplicons, regions flanking the target genes were cloned, and the PCR amplified products were purified and quantified using Nanodrop spectrophotometry providing an estimate of equivalent copy number. Serial dilutions from each standard were run in triplicate to produce a standard curve used to estimate titres in experimental samples.

The presence of *Wolbachia* in *G. pallidipes* was assessed initially with the *Wolbachia* surface protein *Wsp* gene primer set and cycling conditions of Jeyaprakash and Hoy [45]. The *ISNew* PCR was performed with only one primer targeting the flanking terminal inverted repeats (TIR) of the insertion sequence element [46]. In response to the lack of detectable amplicons, we conducted an additional highly sensitive *wsp* PCR-blot technique to detect *Wolbachia* in the *G. pallidipes* sample set according to the protocols outlined by Schneider *et al.*, [47].

### Statistical analysis of virus and symbiont copy numbers

Treatments were analysed by Analysis of variance (ANOVA) and individual treatment means compared with the Tukey-Kramer HSD test [48]. Analyses were performed using Excel® 13 (Microsoft Corp.), RExcel [49] and R [50] programs.

## Results

### Dynamics of GpSGHV in recipient adult *G. pallidipes*

**Figure 1:**
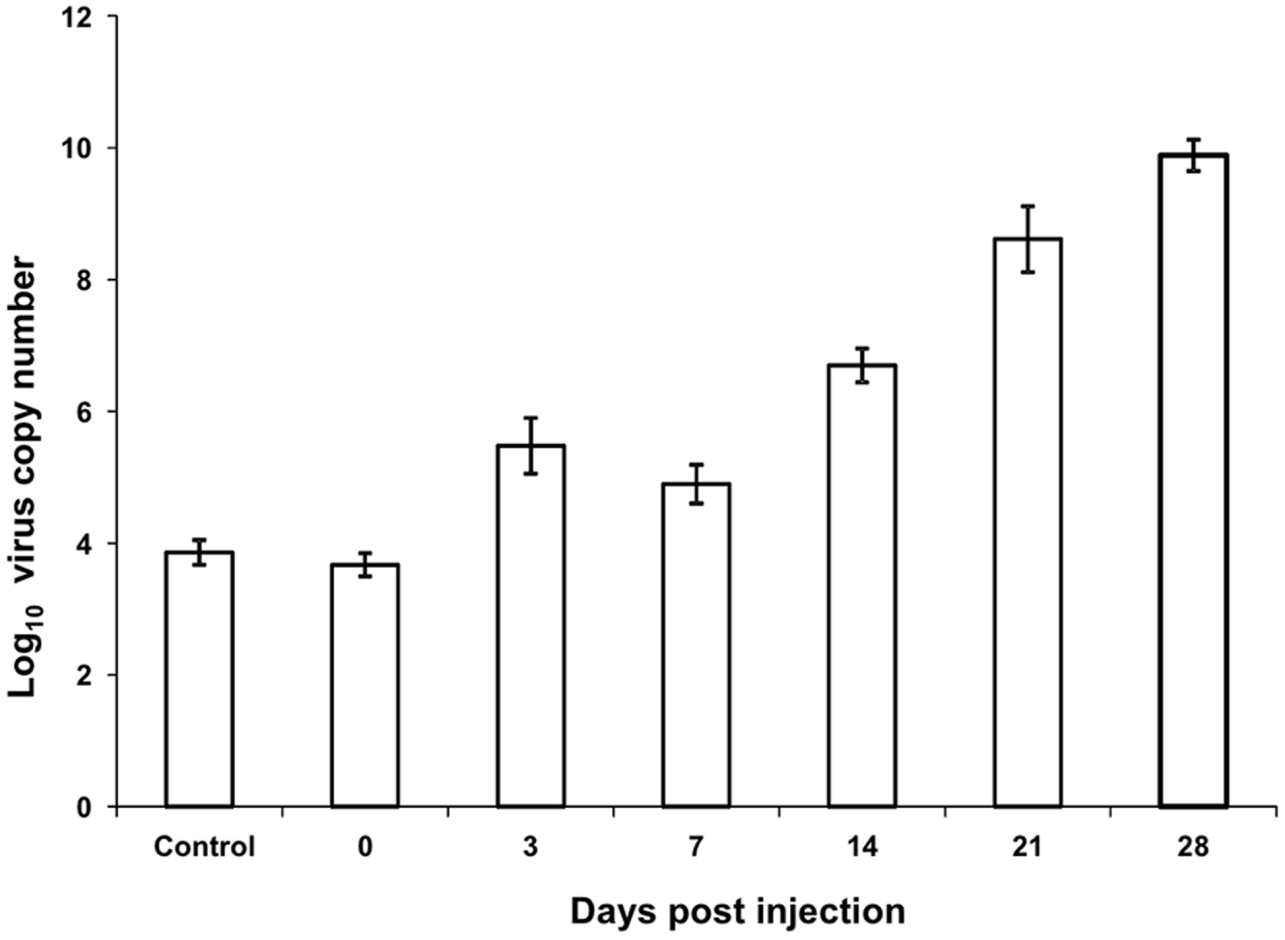
The impact of virus challenge on GpSGHV replication in *G. pallidipes*. The relative virus load in treated *Glossina pallidipes* measured between 0 and 28 days post-injection. Virus load was determined by qPCR on genomic DNA extracted from whole flies using GPSGHV-specific primers. The results were calibrated against a standard curve prepared from known levels of a PCR product that included the smaller qPCR target sequence. The *y*-axis represents the average virus copy numbers per fly. Error bars are indicated.

**Figure 2:**
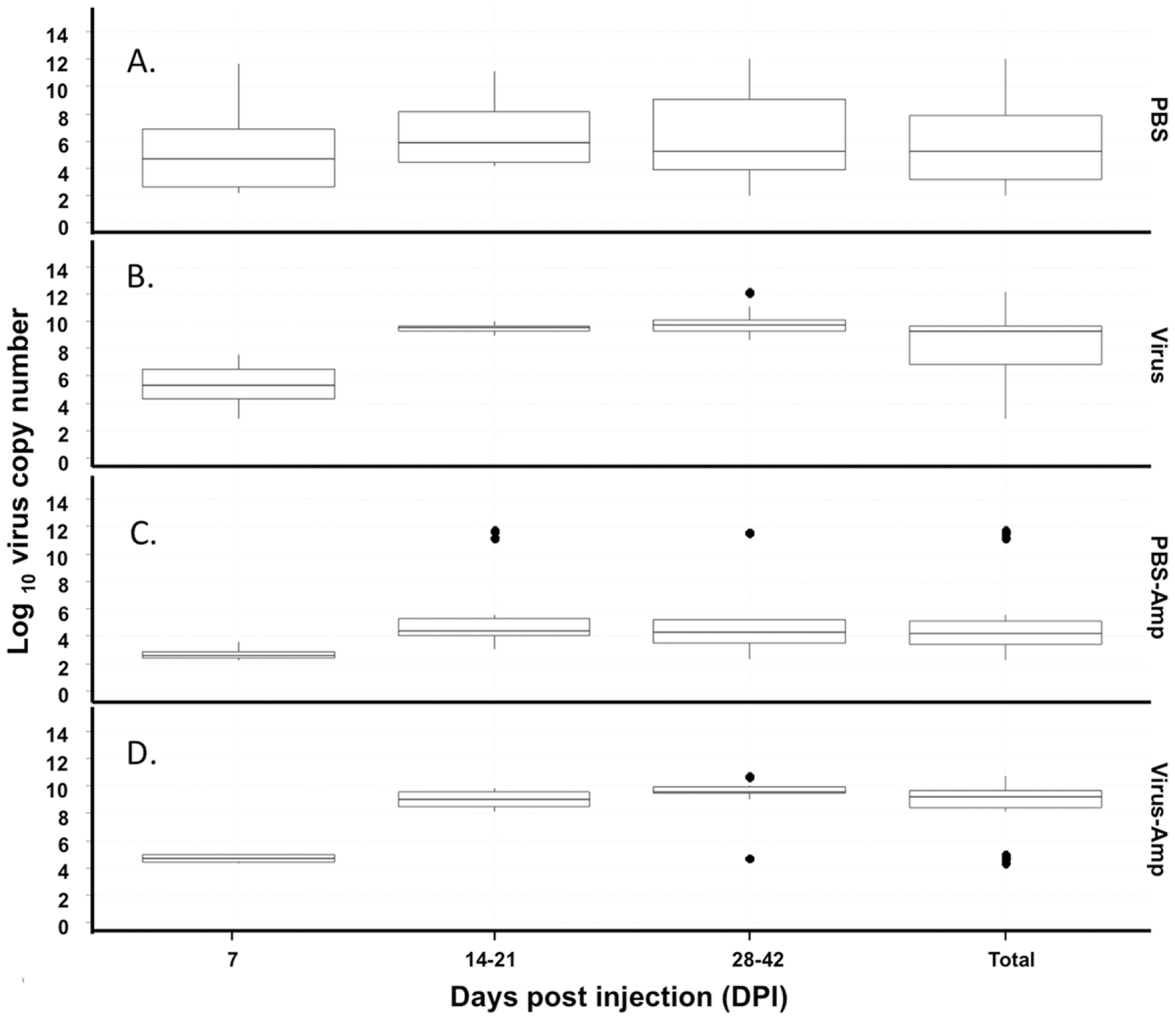
The impacts of virus injection and ampicillin treatment on the virus replication. The virus copy numbers with error bars calculated with qPCR on genomic DNA extracted from whole flies (N = 8) sampled at different intervals post-injection. The different treatments include: (A) the PBS (control)-inoculated flies; (B) virus-inoculated flies; (C) PBS-inoculated flies fed ampicillin amended blood; and (D) virus-inoculated flies fed ampicillin amended blood. Note that the superinfection (B, D) resulted in a significant increase in viral levels within two weeks post- injection. Black dots denote the outliers.

Intrahaemocoelic inoculation of *G. pallidipes* with virus preparation did not cause any initial perturbations in adult activity or fitness. Virus-challenged adults mated, imbibed blood meals and produced numbers of male and female F_1_ progeny during the initial five weeks that mirrored those produced by control (PBS-injected) females (**Table S2**). Surprisingly, haemocoelic injection of the GpSGHV into adults did not increase the incidence of SGH^+^ in those flies (**Table 1**); we expected that bypassing both the cuticle and gut barriers would have induced heavy infections and high incidence of SGH^+^ in virus-injected adults. Time-course assays demonstrated that virus-injected flies had a virus titre (∼10^9^ virus copy number) after 21 dpi that was significantly greater than levels (∼10^4^) detected at 0 dpi (df = 137, F = 224.46, *P*<0.0001) (**Fig. 1**). However, dissection of adults sampled throughout the 49 dpi interval revealed only 1.4% (1/70) and 3.2% (6/186) cases of detectable SGH from control and virus-injected flies, respectively (**Table 1**). The qPCR conducted on DNA samples demonstrated that baseline virus titres in the PBS-injected adults remained relatively stable throughout the duration of the experiment and aging did not correlate to increased viral titres (**Fig. 2A**). Ampicillin treatments, although not impacting the average virus titres of the virus- or PBS-infected flies, reduced the variance when administered to test flies. Excluding the outliers that harboured symptomatic infection (**Fig. 2**), the virus levels varied from 10^4^ to 10^7^ copies in the PBS(control)-injected non-injected flies, values that agreed with the virus loads detected previously in asymptomatic colonized *G. pallidipes* [42]. It should be noted that those few control and virus-injected adult flies displaying symptomatic infections (outliers) harboured similar high levels of virus copies (**Table 1**). Significantly, virus-injected female flies sampled at very late intervals stages (60 dpi) and known to have produced F_1_ adults displaying SGH also did not display SGH symptoms (data not shown). Unlike the PBS-control flies, the titres in the virus-injected flies (excluding those diagnosed as symptomatic) increased to >10^9^ copies per fly over the seven-week sampling interval and were significantly greater (*P*<0.0001) than the levels associated with the PBS-injected asymptomatic flies (**Fig. 2A, B**).

**Table 1:** Summary of the incidence of salivary gland hypertrophy (SGH^+^) symptoms in the *G. pallidipes* parents from the different treatments.

Treatment^a^	Number of flies dissected	SGH^+^ incidence (virus copy number in SGH^+^ individuals)
Control (PBS injected) fed normal blood	70	1 (10^9^)
Virus-injected fed normal blood	186^b^	6 (10^8^–10^11^)
PBS-injected fed ampicillin-amended blood	72	3 (10^8^–10^11^)
Virus-injected fed ampicillin-amended blood	74	2 (10^8^–10^11^)

a ampicillin treatment involved tri-weekly feeding of adult flies on blood supplemented with 40 µg ampicillin per ml. Groups of adults (4–6) were sampled from the adult cages at 6–8 day intervals over a 49-day period.

b These numbers also include flies sampled from the two additional virus injected cages.

**Figure 3:**
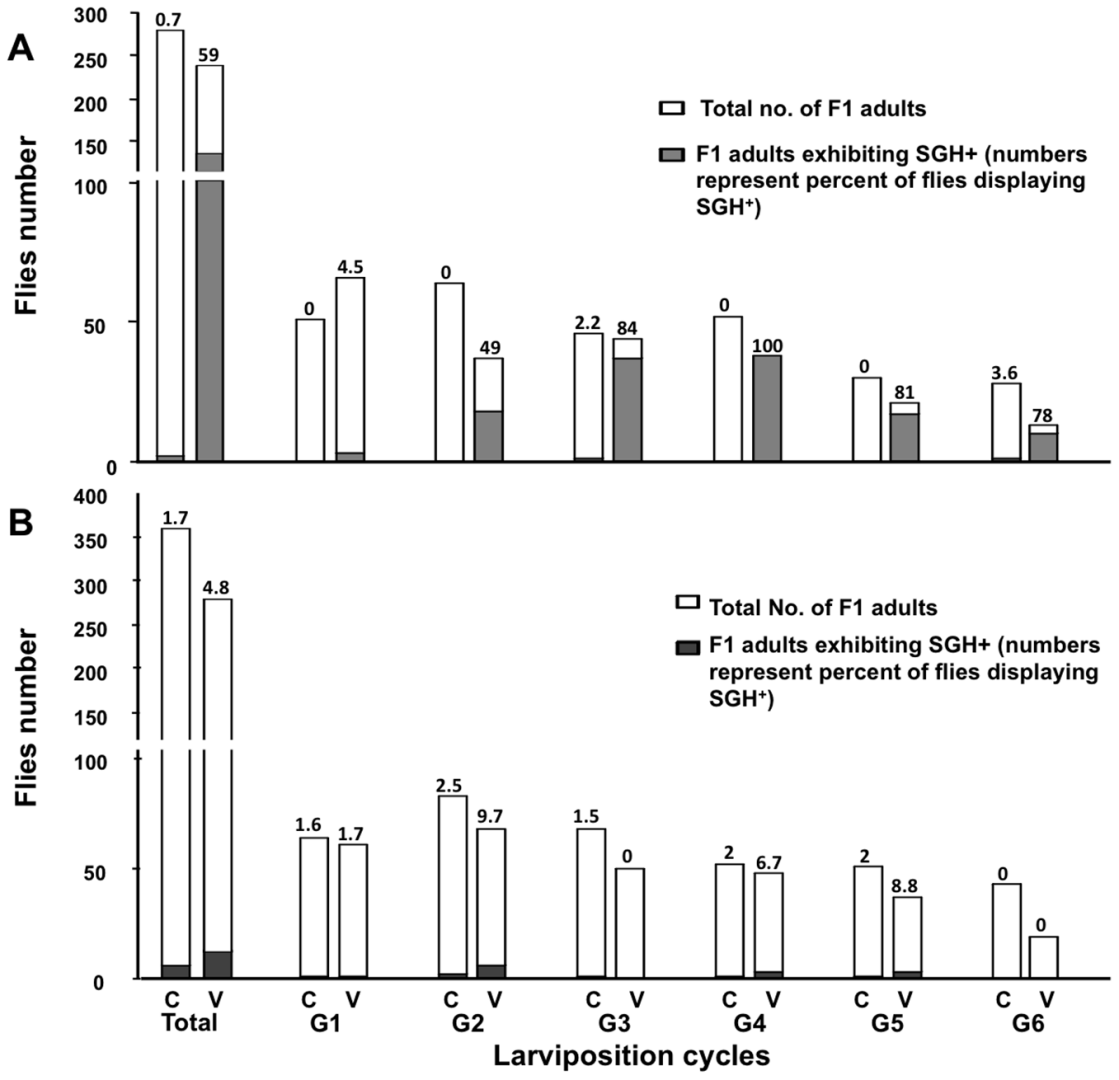
Incidence of symptomatic infections in challenged flies. Number of F_1_ adults displaying SGH^+^ produced from PBS (C)- and virus (V)-injected parents fed normal (panel A) and ampicillin-supplemented (panel B) blood meals. The different larviposition (G) cycles of the F_1_ generation are indicated in the *x*-axis, while the numbers of flies analyzed are shown on the *y*-axis. The numbers above the bars represent the percentage of flies exhibiting the SGH+ syndrome.

Virus-injected and PBS-injected flies, containing ∼10^9^ and ∼10^2−4^ virus copies per fly, respectively, (**Fig. 1**), released similar levels (∼10^2^) of virus copies into the blood during a single feeding event (**Fig. S1**). In contrast, flies displaying SGH^+^ symptoms produce and release large numbers of enveloped virus in the lumen of the salivary gland, depositing up to 10^7^ virus copies into the blood per feeding event [16]. Our data suggests that if replication occurs in the salivary glands of the asymptomatic virus-injected adults, it is only at a low level resulting in negligible release of virus particles during membrane feeding. Virus inoculation, although not inducing increased levels of SGH^+^, induced a chronic disease resulting in a marked reduction in fly survival at the late stages (>35 dpi); the increased mortality rates resulted in reductions in pupal production (**Table S2,** **Fig. 3**).

### Impact of GpSGHV inoculation on F_1_ progeny

The relative duration of pregnancies and size of third instar larvae, length of pupal period, adult eclosion rates, and sex ratio of F_1_ adults for virus-infected treatment were similar to those observed with the control treatment (**Table S2**). In replicated assays, the frequency of SGH^+^ symptoms in the F_1_ progeny produced at different larviposition cycles was determined (**Fig. 3A**). In control assays (N = 271) only two F_1_ adults (0.7%) displayed detectable SGH^+^ symptoms whereas 59% of the F_1_ adults produced by virus-injected females exhibited SGH^+^ symptoms. It should be noted that the F_1_ adults were dissected within 0–24 h post-eclosion, suggesting that the GpSGHV infected and replicated in salivary glands during the differentiation of imaginal discs in pupae. The prevalence of SGH^+^ in these newly emerged F_1_ adults was associated with their larviposition cycle, SGH^+^ symptoms increased from 4.5% in adults from the first larviposition cycle to 100% in adults from the fourth larviposition cycle (**Fig. 3A**). The ability of GpSGHV-injected females to induce high levels of SGH^+^ symptoms in the F_1_ adults correlated with increases in viral titre in the parental generation; the F_1_ adults from the third larviposition cycle were produced by mothers at 28–42 dpi that contained ∼10^9^ viral copies/fly (**Fig. 2B**). Therefore, we propose that virus injection induces virus titres in adults that result in sufficient trans-generational transfer of virus to induce SGH^+^ symptoms during F_1_ adult development.

Ten pairs of F_1_ females and males emerging from the fifth larviposition cycle, sampled from both control and virus-injected treatments, copulated readily when placed together in small cages. It should be noted that at this cycle we estimated >80% of the F_1_ from the virus-injected parents would be SGH^+^. The mating pairs established from the F_1_ adults from the asymptomatic PBS-injected adult flies, produced progeny at 10–14 day intervals, whereas the F_1_ adults (5^th^ larviposition cycle) from virus-injected parents, presumed to be SGH-positive at emergence, produced no progeny.

### The presence of *Sodalis* and *Wigglesworthia* and the absence of *Wolbachia* in *G. pallidipes*

**Figure 4:**
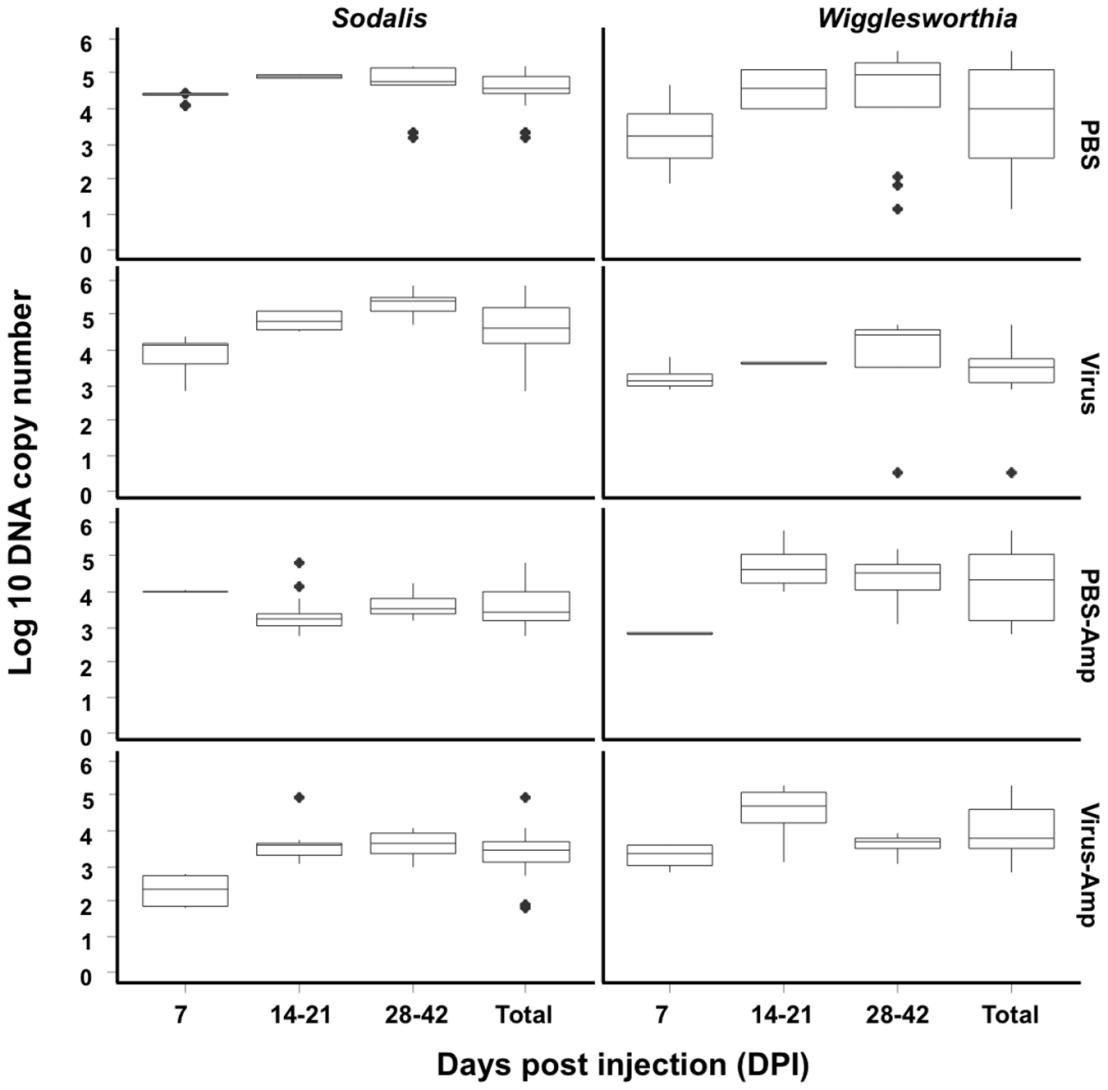
The impact of virus injection and ampicillin treatment on the *Sodalis* and *Wigglesworthia*. The relative copy number of *Sodalis* and *Wigglesworthia* in the F_1_ adults detected with qPCR on genomic DNA extracted from teneral flies (N = 8) sampled at different intervals post injection. Blak dots are the outlier.

**Figure 5:**
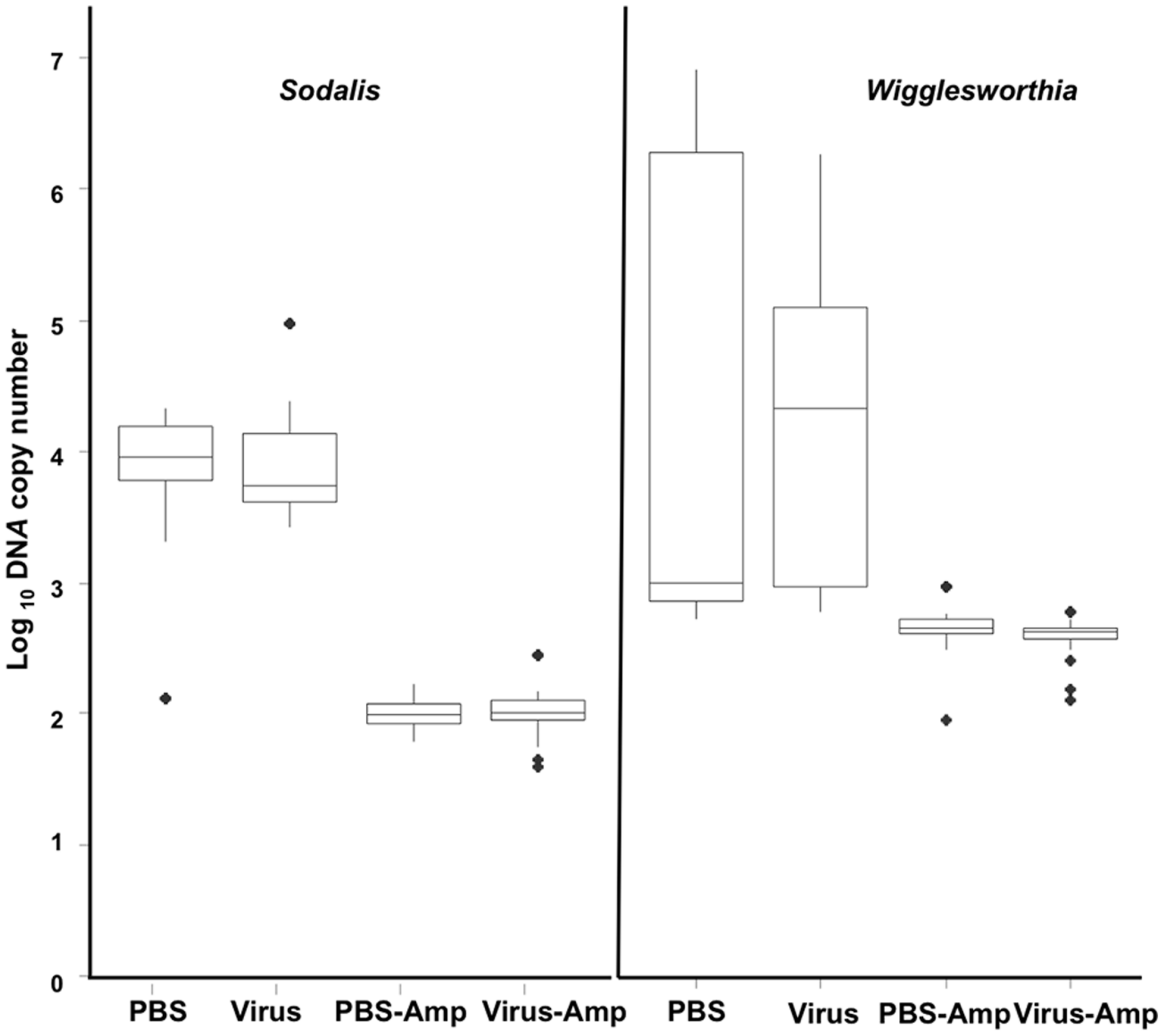
The impact of treatments on *Sodalis* and *Wigglesworthia* titres in the F_1_ generation of *G. pallidipes*. The relative titres of *Sodalis* and *Wigglesworthia* in the F_1_ progeny from both PBS-control and virus-injected treatments fed normal and ampicillin (Amp) supplemented blood meals. DNA copy numbers estimated by qPCR conducted on genomic DNA extracted from teneral flies (N = 8). Ampicillin treatment caused dramatic reductions in bacterial levels in the teneral F_1_ progeny. Black dots denote outliers.

The *G. pallidipes* lab colony used in our studies, like other tsetse flies [51], was expected to harbour species of both *Sodalis* and *Wigglesworthia* and possibly *Wolbachia*. Initial comparisons among samples from PBS (control)- and virus-injected *G. pallidipes*, using both conventional PCR and qPCR, demonstrated that lab colony flies retained a similar titre (1–5×10^5^ copies/fly) of *Sodalis* and *Wigglesworthia* throughout the adult lifespan (**Fig. 4**). Superinfection with the GpSGHV had no detectable impact on the symbiont titer in parent flies; in both treatments bacterial levels averaged 5×10^3^ cells/fly at seven days and increased to 1×10^5^ cells per fly by 21 days post injection. The newly eclosed F_1_ male and female progeny sampled from the different larviposition cycles from both PBS- and virus-injected treatments (**Fig. 5**) contained ∼five- to ten-fold fewer copies (F = 53.76; p<0.0001) of these symbionts than detected in parents (**compare with** **Fig. 4**).

Different primers were employed to screen for *Wolbachia* infection in *G. pallidipes*; the first primer set targeting the single copy *Wolbachia* outer surface protein gene (*wsp*) and the second targeting a high copy number transposon locus, the insertion sequence element *New* (*ISNew*) [52]. However, no amplicons were produced using control DNA with these PCR reactions, suggesting the complete absence of *Wolbachia* in the *G. pallidipes* colony (**Fig. S2**). Subsequently we employed a highly sensitive *wsp* PCR-blot technique to reveal potential low titre *Wolbachia* infections that might have been overlooked with *wsp* and *ISNew* PCR. Negative results suggested that the lab colony is either void of *Wolbachia* or harbours the symbiont at levels far below the detection limit of even our high-sensitivity screening approach. In light of negative results with control *G. pallidipes* additional qPCR screening of *Wolbachia* in virus-injected and ampicillin treated samples were not conducted.

### Impact of ampicillin on *Sodalis* and *Wigglesworthia*

Groups of newly emerged *G. pallidipes* injected with either PBS or virus gland suspension were fed blood three times per week supplemented with 40 µg ampicillin/ml of blood throughout adulthood. Ampicillin treatments caused a significant 10-fold reduction in *Sodalis* titres in *G. pallidipes* (**Fig. 4**). However, the dosage of ampicillin given to these flies did not totally clear *Sodalis*; the reduced levels of *Sodalis* observed after six blood meals remained constant throughout the parental sampling period. Unlike *Sodalis*, the titre of *Wigglesworthia* in flies injected with either PBS or the virus gland suspension were not impacted significantly by being fed ampicillin amended blood (**Fig. 4**); *Wigglesworthia* titres in the ampicillin treatments increased with fly age from ∼5×10^3^ to 1×10^5^ cells per fly.

The ampicillin-induced reduction of *Sodalis* in the parent flies impacted the bacterial levels in the F1 progeny. Compared to flies fed non-supplemented blood, male and female progeny from either ampicillin-treated PBS- or virus-injected adults, regardless of the larviposition cycle, lacked detectable *Sodalis* amplicons using conventional PCR reactions. Similarly, ampicillin treatment, although not suppressing *Wigglesworthia* titres in the parent generation, suppressed titres in the F1 progeny., qPCR demonstrated that genomic DNA from the F_1_ of ampicillin-treated parents contained ∼10^2^ *Sodalis* and 2.7×10^2^ *Wigglesworthia* copies/fly, titres that were significantly lower than those found in the progeny from their non-antibiotic treated counterpart adults (**Fig. 5**). Potentially, the antibiotic treatment cleared extracellular *Sodalis* and *Wigglesworthia* without impacting intracellular *Sodalis* infecting stem cells. It should be noted that the copy numbers detected in the F_1_ progeny of ampicillin treatments are close to the detection limit of the qPCR suggesting that the F_1_ progeny are likely void of *Sodalis* and *Wigglesworthia*.

### Impact of ampicillin treatments on the *G. pallidipes* and GpSGHV

The ampicillin therapy, although dramatically lowering the bacterial symbiont levels, did not cause drastic impacts on the survival, reproductive behaviour, or fertility of treated *G. pallidipes* adults (**Table S2**). Flies fed ampicillin treated blood readily mated and produced a normal complement of F_1_ offspring. Antibiotic treatment caused a minor increase in pupal mortality in the F_1_ from both the PBS-controls (5%) and the virus-injected adults (8%). However, these impacts were much less than ∼20% reduction in emergence observed in an earlier study with the ampicillin treatments of *G. m. morsitans* [30]. Importantly, compared to the GpSGHV levels in adults fed control blood, the ampicillin treatment did not significantly change GpSGHV titres in either the PBS- or virus-injected adults (*P* = 0.598; **Fig. 2**).

**Figure 6:**
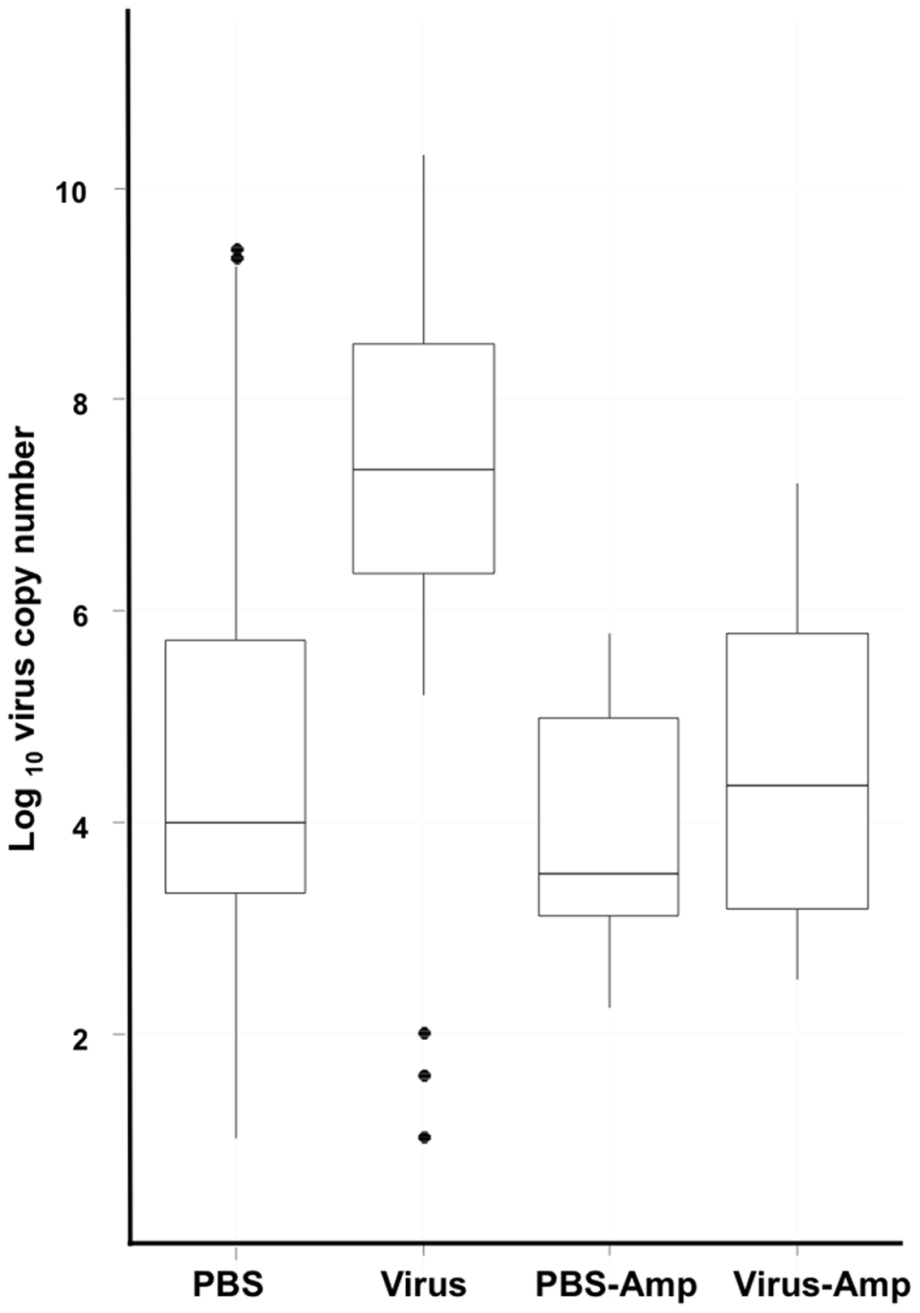
Ampicillin treatment reduces the virus load in F_1_ progeny of superinfected parents. Virus copy numbers detected by qPCR on genomic DNA extracted from the F_1_ progeny (N = 8) of superinfected parents (Virus) were significantly greater than levels detected in the F_1_ progeny of asymptomatic control (PBS) flies. Feeding the superinfected parents ampicillin-amended blood meals (Virus-Amp) reduced virus copy numbers to levels similar to those detected in the asymptomatic PBS (control) and PBS-Amp flies. Black dots denote outliers.

Most notable is our finding that antibiotic treatment negated the expression of SGH^+^ symptoms in the F_1_ progeny of the virus-injected parents (**Fig. 3B**). Virus titres in the ampicillin-treated, virus-injected parents were comparable to those titres detected in the virus-injected adults fed untreated blood (**Fig. 2**). However, the virus titres (5×10^4^ copies/fly) of the F_1_ progeny from the antibiotic treated virus-injected parents were similar to the virus titres (1×10^4^ copies/fly) detected in the F_1_ progeny from the symbiotic PBS-injected controls (**Fig. 6**). These levels were significantly less than the virus titres (∼4×10^7^ copies/fly) detected in the F_1_ progeny from virus-injected parents fed unamended blood. Therefore, removal of the symbiome either suppressed the trans-generational transfer of the virus via the milk glands to the F_1_ or blocked the ability of the SGHV to infect and replicate in the salivary gland of the F_1_ adults.

## Discussion

In this study, we confirmed that the *G. pallidipes* colony universally harbours an asymptomatic GpSGHV infection. The virus levels and the low incidence of SGH^+^ detected in the control treatments are similar to the background levels observed routinely in this *G. pallidipes* colony [14], [16]. Injection of virus derived from gland homogenates into asymptomatic flies resulted in increased virus titres but did not trigger increased incidence of SGH^+^ symptoms. Evidently, the virus was able to infect and undergo limited replication in cells that were not infected by the virus present in asymptomatic flies. It is likely that haemocoelic injection provided the virus access to tissues and cells that did not harbour the latent virus present in the asymptomatic fly. Potentially, the asymptomatic infection may provide cross-protection limiting the development of SGH^+^ symptoms in virus-injected flies. Previously, Jura et al [53] and Sang et al [54] reported that injecting newly larviposited third-instar *G. m. morsitans* and *G. m. centralis* with GpSGHV-infected gland homogenate resulted in the production of 100% symptomatic adults. These reports, in combination with our observations on *G. pallidipes*, suggest that the GpSGHV is capable of infecting and replicating during the ontogeny of the adult salivary gland occurring during pupation, but it is unable to develop SGH^+^ symptoms in the differentiated adult salivary gland cells in a host-dependent manner. It should be noted that, the results obtained with GpSGHV in *G. pallidipes* differed markedly from those achieved by injecting adult house flies with the MdSGHV. In house flies, haemocoelic injection with very low levels of virus induced both a 100% incidence of SGH^+^ symptoms and a total shutdown of egg production within 72–96 h post-injection [55], [56].

The variable but low virus titres in control *G. pallidipes* suggest that the asymptomatic or latent infection is restricted spatially and temporally to select cells or undergoes only partial replication in adult cells to maintain titres at steady-state levels throughout the one- to three-month adult stage. Presently, nothing is known about the replicative pathway of GpSGHV in these asymptomatic tsetse flies. Potentially, observed changes in viral copy number represent only DNA replication and may not involve the production of infectious viral particles. Unlike the situation with GpSGHV, the housefly-MdSGHV system does not harbour asymptomatic infections, rather, the virus, once present in the host rapidly induces SGH^+^ symptoms leading to a massive increase in viral titre [57].

The dynamics of GpSGHV replication in virus-challenged *G. pallidipes* flies differ from MdSGHV injected into house flies [57]. Injected MdSGHV reached titres in excess of 10^10^ copies/fly in its homologous host (house fly) and 10^9^ copies/fly in a heterologous host (*Stomoxys calcitrans*), respectively, by two dpi [56]. In house flies, the increased viral titre can be explained by viral development in the salivary gland cells; by three dpi this tissue displayed fully developed SGH^+^ due to massive viral replication [58]. However, *S. calcitrans* challenged with MdSGHV did not develop SGH^+^ symptoms, yet still supported relatively rapid replication of MdSGHV. The subsequent consequence of the MdSGHV replication in both hosts was virus-induced sterility in female flies. Injection of GpSGHV into *G. pallidipes* did not block oogenesis or intra-uterine larval development.

The F_1_ SGH^+^ adults prepared as single pairs were observed to mate readily in small cages. These observations differed from the mating behaviour exhibited by MdSGHV-infected female house flies; these females refused to copulate and rejected both SGH^+^ and healthy males [55]. However, the mating's of F_1_ SGH^+^ *G. pallidipes* did not produce any progeny; such virus-induced sterility is believed to be responsible for virus-induced colony collapse in tsetse factories and agrees with prior reports conducted on symptomatic *G. pallidipes* and *G. m. centralis* [16], [54], [59].

The additional viral replication, induced by injection with the infected gland preparation, did not hinder either the maintenance of either *Sodalis* or *Wigglesworthia* in the parent flies or the subsequent transfer of these bacteria to F_1_ progeny. The titres detected in adult *G. pallidipes* are similar to bacterial levels detected in *G. m. morsitans* [60]. The relatively low titres of *Sodalis* detected in the newly emerged F_1_ adults suggest that replication of the free-living *Sodalis* during pupation is suppressed; bacteria deposited *in utero* were incubated for approximately thirty days at 24°C after larviposition yet they attained titres of less than 10^4^ cells/adult at eclosion. The intracellular *Wigglesworthia* symbiont also displayed lower titres in teneral F_1_ adults.

Antibiotic therapy of *G. pallidipes* did not impact their fecundity or the rate of F_1_ adult eclosion. Similar results have been observed with *G. m. morsitans* fed ampicillin-treated blood meals [30], [61]. Furthermore, antibiotic treatment of asymptomatically infected (PBS-injected) flies did not suppress GpSGHV titres in their respective F_1_ progeny; virus levels in these insects were comparable to the F_1_ progeny from PBS-injected adults fed normal blood. These findings conflict somewhat with Wang *et al.*, [21] who reported that antibiotic treatment of asymptomatic *G. m. morsitans* resulted in a significant suppression of viral copy numbers in the F_1_ and F_2_ generation. It should be noted that analyses on *G. m. moristans* were conducted on flies collected at forty days post-eclosion; in our study the virus titration was conducted on teneral F_1_ flies. Secondly, the *G. m. moristans* colony does not express the symptomatic SGH^+^ phenotype that is associated, albeit at low levels, with the *G. pallidipes* colony.

The lack of detectable *Wolbachia* in *G. pallidipes* is in agreement with past reports. In their large-scale screen on diverse *Glossina* species, Doudoumis *et al.*, [22] showed that only 1.2% (22/1896) of all tested *G. pallidipes* were positive for *Wolbachia* infection. In this study, more cases of infection were found in field strains than in lab colonies. In accordance with our findings they reported that samples from the *G. pallidipes* lab colony in Seibersdorf, Austria, were all negative for *Wolbachia*. Whether the lack of *Wolbachia* in the *G. pallidipes* is linked to the asymptomatic maintenance or the harmful impact of symptomatic infection on GpSGHV in this colony is unknown. In the light of the recently published report demonstrating the antiviral activity induced by *Wolbachia* in infected hosts, [62] the absence of *Wolbachia* in the *G. pallidipes* colony may explain the severe negative impact of the GpSGHV on large scale colonies. Although the virus was reported in other tsetse fly species harbouring *Wolbachia* infection [63], no harmful impact of the virus on mass rearing has been reported. A recent study of *G. fuscipes fuscipes* populations suggested that the levels of PCR-detectable GpSGHV is influenced by the tsetse fly genotype and is inversely correlated with the prevalence of *Wolbachia* [64].

Continuous exposure of *G. pallidipes* to ampicillin-treated blood was expected to cause a partially decrease the *Sodalis* titres without impacting *Wigglesworthia* [30]. Evidently, this antibiotic is unable to either access and/or kills the intracellular phenotypes. Therefore, the ampicillin therapy was able to clear the exocellular bacteria associated with the milk gland secretions preventing subsequent transmission to the F_1_ progeny. Antibiotic treatment and concomitant reduction of bacterial symbiont levels in the F_1_ of the virus-injected parents was correlated with suppression of the SGH^+^ phenotype in the F_1_. This finding was surprising as microbial symbionts have been reported frequently to prime the insect immune system increasing host resistance to infection by various viruses, protozoans, and nematodes [32], [34], [62], [65]. In the case of tsetse flies, the bacterial associates at several levels have been reported to modulate host innate defences. For example, the *Wigglesworthia* associated with *G. m. morsitans* adults stimulates the production of the catalytically active tsetse fly peptidoglycan recognition protein (PGRP-LB) in the midgut bacteriome [32], [66]. This enzyme scavenges the peptidoglycan fragments produced by *Wigglesworthia* preventing activation of the innate defence systems that can impact tsetse fly fitness and reduce fecundity [38]. An important outcome is that antibiotic-treated flies produce F_1_ that are highly sensitive to trypanosome infection [30]. However, in the case of *G. pallidipes*, suppression of the symbionts did not impact virus titres in the parental generation suggesting that GpSGHV is unresponsive to ampicillin-induced alterations of the innate defence system.

The concept that symbionts may modulate viral biology in insects is not novel; endosymbionts have been shown to mediate the vector competence of both aphids and whiteflies [67], [68], [68]–[73]. Significantly, treating aphids with tetracycline suppressed GroEL gene expression and reduced potato leaf-roll virus PLRV transmission by 70%. Similarly, Morin *et al.*, [67], [72] provided evidence that the transmission efficiency of the tomato yellow leaf curl virus (TYLCV) by the white fly *Bemisia tabaci* involved endosymbiont-produced GroEL homologues. Feeding white flies anti-GroEL antibodies caused an 80% reduction in TYLC transmission and reduced haemolymph virus titres below detection levels. More recently, Gottlieb *et al.*, [74] demonstrated the GroEL protein produced by the endosymbiont *Hamiltonella* is responsible for the efficient TYLCV transmission by the whitefly B biotype; the Q-biotype lacking the *Hamiltonella* is a less efficient TYLCV transmitter. The interaction of the GroEL with PLRV has been proposed to protect haemolymph-borne virus from host defences allowing for virus transfer to the salivary gland lumen [68], [69], [74]. The primary *Wigglesworthia* and secondary *Sodalis* symbionts of *G. m. morsitans* also produce 60 kDa GroEL chaperones [75]. In the midgut, the *Wigglesworthia* GroEL is the most highly expressed protein, yet remains localized within the bacteriome that harbours the primary symbionts. To date, nothing is known about the distribution and function of the *Sodalis* GroEL. Notably, this secondary symbiont is found in both extracellular and intracellular locations and is not restricted to a bacteriome-type structure [19], [25].

Recent proteomic analysis of GpSGHV revealed that the viral envelope, in addition to containing peptides associated with viral proteins, contained many peptides that displayed homology to host proteins and to bacterial symbionts [13]. Significantly, a series of *Sodalis* proteins including the major outer membrane lipoprotein, outer membrane protein A, outer membrane protein F, hypothetical phage protein, peptidoglycan-associated lipoprotein, type-III secretion apparatus, and the GroEL peptide were detected in the envelope fraction. These peptides were not detected in the extracted nucleocapsid or asymptomatic salivary gland homogenate fractions. The virus was purified from dissected hypertrophied salivary glands from symptomatic flies and may not represent the phenotype present in the haemolymph or in the milk glands. Regardless, the selective sequestration of these bacterial peptides in the viral envelope suggests a functional role in possible immune evasion or in selective transmission of the virus from infected to healthy flies. Antibiotic treatment would, most likely, reduce the levels of bacterial products available for decorating the viral envelope.

In summary, the GpSGHV in *G. pallidipes* is found typically as a latent infection causing little or no fitness cost to the host fly. The symptomatic state is normally activated in a small percentage of the colony. Attempts to induce SGH^+^ by injecting flies with a high dosage of virus failed but caused increased viral titres. These females after their third larviposition produced F_1_ adults that frequently displayed SGH^+^. Treating parents with antibiotics blocked the trans-generational transmission of the virus that resulted in SGH^+^. The antibiotic therapy suppressed the primary and secondary bacterial symbionts levels in both parent and their subsequent transfer to the F_1_ flies. These findings suggest that this dsDNA insect virus has evolved in close association with the symbiome to mediate the switch from the asymptomatic (latent) to symptomatic state.

## Supporting Information

Table S1 Primers. A list of the primers used to conduct both end-point and quantitative PCR reactions.
(DOCX)

Table S2 Fitness data. Summary of fitness data on the pregnancies and resulting F_1_ progeny from the various treatments.
(DOCX)

Figure S1 **GpSGHV copies released during feeding.** Graph depicting the average number virus copies estimated by qPCR deposited by the *G. pallidipes* adults that have been injected with GpSGHV or with PBS (control) into the blood during a single membrane feeding event. Panel A represents the level of virus in the host fly at the time of feeding (28 dpi) and Panel B the number of virus copies released into the blood during the feeding event from adults harboring low and high levels of GpSGHV copy numbers. It should be noted that unlike these symptomatic flies harboring symptomatic SHG^+^ flies have been reported to release 10^7^ virus copies per feeding event (Abd-Alla et al., 2010).
(DOCX)

Figure S2 **Lack of** ***Wolbachia*** **in** ***G. pallipides*** **females.** *Wolbachia*-specific PCR on *G. pallidipes* females. Presence of *Wolbachia* was tested using two primer sets targeting (**A**) the *Wolbachia* outer surface protein gene (*wsp*), and (**B**) the insertion sequence element *ISNew*. (**C**) Quality of DNA was assessed by using *Glossina*-specific *tubulin* primer (Caljon et al. 2009). *Wsp* and *ISNew* PCR do not produce amplicons in all samples except for the positive control (**A, B**). *Tubulin* PCR, however, shows bright signals in all samples (**C**). Positive controls for PCR are *Wolbachia* high titer *G. morsitans centralis* denoted with (+), not that both gene targets were detected with this DNA template. The (M) designates the 1 kb DNA ladder used as size reference.
(DOCX)
