# Supplementary material for: Transgenerational Transmission of the Glossina pallidipes Hytrosavirus Depends on the Presence of a Functional Symbiome
Source: PLoS One. 2013 Apr 22;8(4):e61150. doi: 10.1371/journal.pone.0061150 (PMC3632566; doi:10.1371/journal.pone.0061150)
Supplement: Table S1 — Primers. A list of the primers used to conduct both end-point and quantitative PCR reactions. (DOCX) [file pone.0061150.s001.docx]

**Table S1**. **Primers.**  A list of the primers used to conduct both end-point and quantitative PCR reactions.

| **Primer** | **Target gene** | **Primer sequence** | **Reference** |
| --- | --- | --- | --- |
| qPCRFwd^a^  qPCRRev^a^ | SGHV ORF005  (*odv-e66*) | 5'- CAAATGATCCGTCGTGGTAGAA - 3'  5'- AAGCCGATTATGTCATGGAAGG -3' | [1] |
| GpSGHVfwd ^b^  GpSGHVrev ^b^ | SGHV ORF005  (*odv-e66*) | 5'- GCTTCAGCATATTATTCCGAACATAC -3'  5'- GATCCTGCTCGCGTAAACCA -3' | [2] |
| Sod-FliCF ^b^  Sod-FliCR ^b^ | *FliC* (flagellin) | 5'- GCA GTT TCA GGA TAC CC -3'  5'- GGC GGA AAA TGG TAT AG -3' | [3] |
| sodqPCR-FliCF^a^  sodqPCR-FliCR^a^ | *FliC* (flagellin) | 5'- GAA GCC ACC GAT CCT GTA AC -3'  5'- CAT CTT TGC CCG TAG AAA TCA C -3' | [4] |
| WiggthiCF ^b^  WiggthiCR ^b^ | *thiC* (thiamine biosynthesis gene) | 5'- GAACAAGCAATACAAGGTGTAG-3'  5'- CTATCG GTACATGTCCAGGTCCTTC-3' | this paper |
| WiggqPCRthiCF^a^  WiggqPCRthiCR^a^ | *thiC* (thiamine biosynthesis gene) | 5'-GACATCAAATCGCGTTACTGG -3'  5'- GACTTGTACGTGATATTTCCAAGC-3' | this paper |
| Tsetse-tubulinF  Tsetse-tubulinR | *β-tubulin* | 5'- GAT GGT CAA GTG CGA TCC T -3'  5'- TGA GAA CTC GCC TTC TTC C -3' | [5] |
| Wsp fwd  Wsp rev | *wsp* (surface protein gene) | 5'-TGGTCCAATAAGTGATGAAGAAACTAG  CTA-3'  5'-AAAAATTAAACGCTACTCCAGCTTCTGC  AC -3' | [6] |
| ISNew TIR | *IS* (insertion sequence element) | 5’-GGC TTT GTT GCA TCG C-3’ | [7, 8] |

^a^ denotes primers used for qPCR reactions

^b^ denotes primers used to prepare standard PCR products

References

1. Abd-Alla A, Cousserans F, Parker A, Bergoin M, Chiraz J et al. (2009) Quantitative PCR analysis of the salivary gland hypertrophy virus (GpSGHV) in a laboratory colony of *Glossina pallidipes*. Virus Res 139: 48-53. doi:10.1016/j.virusres.2008.10.006

2. Kariithi HM, Ahmadi M, Parker AG, Franz G, Ros VID et al. (2012) Prevalence and genetic variation of salivary gland hypertrophy virus in wild populations of the tsetse fly *Glossina pallidipes* from southern and eastern Africa. J Invertebr Pathol doi: 10.1016/j.jip.2012.04.016

3. Toh H, Weiss BL, Perkin SA, Yamashita A, Oshima K et al. (2006) Massive genome erosion and functional adaptations provide insights into the symbiotic lifestyle of *Sodalis glossinidius* in the tsetse host. Genome Res 16: 149-156. PM:16365377

4. Weiss BL, Maltz M, Aksoy S (2012) Obligate symbionts activate immune system development in the tsetse fly. J Immunol 188: 3395-3403.

5. Caljon G, Broos K, De G, I, De RK, Sternberg JM et al. (2009) Identification of a functional Antigen5-related allergen in the saliva of a blood feeding insect, the tsetse fly. Insect Biochem Mol Biol 39: 332-341. PM:19507303

6. Jeyaprakash A, Hoy MA (2000) Long PCR improves *Wolbachia* DNA amplification: *wsp* sequences found in 76% of sixty-three arthropod species. Insect Mol Biol 9: 393-405. PM:10971717

7. Schneider DI, Garschall KI, Parker AG, bd-Alla AM, Miller WJ (2012) Global *Wolbachia* prevalence, titer fluctuations and their potential of causing cytoplasmic incompatibilities in tsetse flies and hybrids of *Glossina morsitans* subgroup species. J Invertebr Pathol <http://dx.doi.org/10.1016/j.jip.2012.03.024> PM:22516306

8. Wu M, Sun LV, Vamathevan J, Riegler M, Deboy R et al. (2004) Phylogenomics of the reproductive parasite *Wolbachia pipientis w*Mel: a streamlined genome overrun by mobile genetic elements. PLoS Biol 2: E69. PM:15024419
