# Supplementary material for: Transgenerational Transmission of the Glossina pallidipes Hytrosavirus Depends on the Presence of a Functional Symbiome
Source: PLoS One. 2013 Apr 22;8(4):e61150. doi: 10.1371/journal.pone.0061150 (PMC3632566; doi:10.1371/journal.pone.0061150)
Supplement: Table S2 — Fitness data. Summary of fitness data on the pregnancies and resulting F1 progeny from the various treatments. (DOCX) [file pone.0061150.s002.docx]

| Treatment | Days to first larviposition | Pupal production | | | Total number of pupae | Parental mortality at (60 dpi)^1^ | | Number dead pupae | Days in pupal stage | Adult eclosion rate | Sex ratio (% males) |
| --- | --- | --- | --- | --- | --- | --- | --- | --- | --- | --- | --- |
|  |  | days 1- 35 | | days 36-60 |  |  |  |  |  |  |  |
| **Fed control blood** |  |  |  | |  | |  |  |  |  |  |
| PBS control | 16 | 238 | 161 | | 399 | | 10% | 18 | 33-38 | 94% | 55% |
| Virus Injected | 17 | 178 | 114 | | 296 | | 70% | 12 | 32-37 | 95% | 46% |
| **Fed ampicillin blood** |  |  |  | |  | |  |  |  |  |  |
| PBS control | 16 | 187 | 199 | | 386 | | 11% | 20 | 32-36 | 95% | 52% |
| Virus-injected | 16 | 179 | 124 | | 303 | | 58% | 22 | 33-38 | 91% | 45% |

**Table S2.** **Fitness data.** Summary of fitness data on the pregnancies and resulting F_1_ progeny from the various treatments.

^1^ Not all of the adults emerging from these pupae were examined for SGH symptoms.
