# Supplementary material for: Transgenerational Transmission of the Glossina pallidipes Hytrosavirus Depends on the Presence of a Functional Symbiome
Source: PLoS One. 2013 Apr 22;8(4):e61150. doi: 10.1371/journal.pone.0061150 (PMC3632566; doi:10.1371/journal.pone.0061150)

**Figure S1. GpSGHV copies released during feeding.** Graph depicting the average number virus copies estimated by qPCR deposited by the *G. pallidipes* adults that have been injected with GpSGHV or with PBS (control) into the blood during a single membrane feeding event. Panel A represents the level of virus in the host fly at the time of feeding (28 dpi) and Panel B the number of virus copies released into the blood during the feeding event from adults harboring low and high levels of GpSGHV copy numbers. It should be noted that unlike these symptomatic flies harboring symptomatic SHG^+^ flies have been reported to release 10^7^ virus copies per feeding event (Abd-Alla et al., 2010).


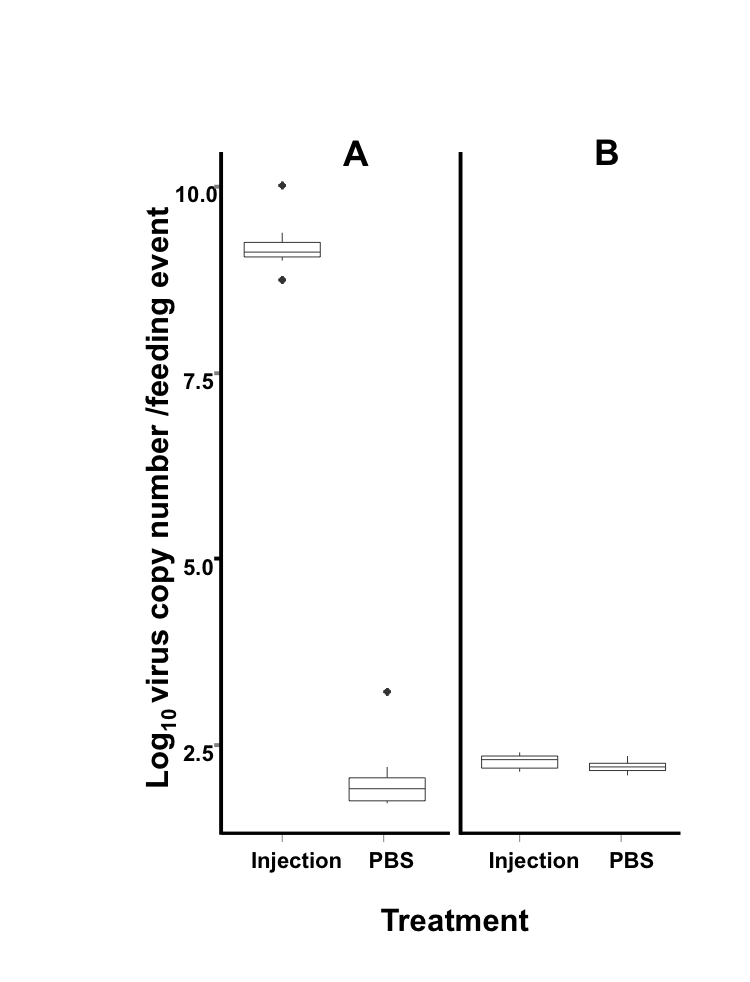

Supplement: Figure S1 — GpSGHV copies released during feeding. Graph depicting the average number virus copies estimated by qPCR deposited by the G. pallidipes adults that have been injected with GpSGHV or with PBS (control) into the blood during a single membrane feeding event. Panel A represents the level of virus in the host fly at the time of feeding (28 dpi) and Panel B the number of virus copies released into the blood during the feeding event from adults harboring low and high levels of GpSGHV copy numbers. It should be noted that unlike these symptomatic flies harboring symptomatic SHG+ flies have been reported to release 107 virus copies per feeding event (Abd-Alla et al., 2010). (DOCX) [file pone.0061150.s003.docx]
