# Supplementary material for: Transgenerational Transmission of the Glossina pallidipes Hytrosavirus Depends on the Presence of a Functional Symbiome
Source: PLoS One. 2013 Apr 22;8(4):e61150. doi: 10.1371/journal.pone.0061150 (PMC3632566; doi:10.1371/journal.pone.0061150)

**Figure S2. Lack of *Wolbachia* in *G. pallipides* females.** *Wolbachia*-specific PCR on *G. pallidipes* females. Presence of *Wolbachia* was tested using two primer sets targeting **(A)** the *Wolbachia* outer surface protein gene (*wsp*), and **(B)** the insertion sequence element *ISNew*. **(C)** Quality of DNA was assessed by using *Glossina*-specific *tubulin* primer (Caljon et al. 2009). *Wsp* and *ISNew* PCR do not produce amplicons in all samples except for the positive control **(A, B)**. *Tubulin* PCR, however, shows bright signals in all samples **(C)**. Positive controls for PCR are *Wolbachia* high titer *G. morsitans centralis* denoted with (+), not that both gene targets were detected with this DNA template. The (M) designates the 1 kb DNA ladder used as size reference.


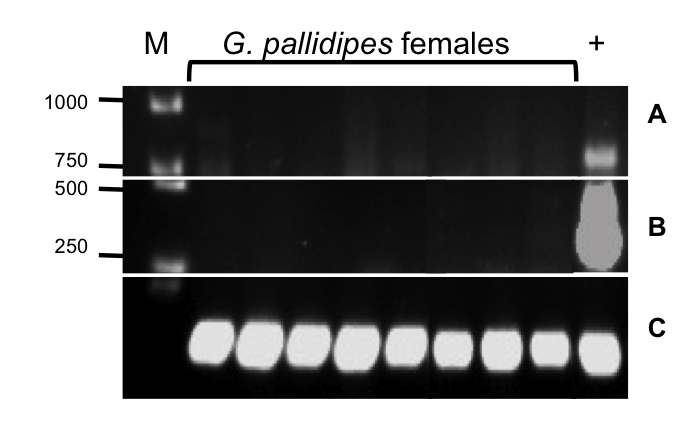

Supplement: Figure S2 — Lack of Wolbachia in G. pallipides females. Wolbachia-specific PCR on G. pallidipes females. Presence of Wolbachia was tested using two primer sets targeting (A) the Wolbachia outer surface protein gene (wsp), and (B) the insertion sequence element ISNew. (C) Quality of DNA was assessed by using Glossina-specific tubulin primer (Caljon et al. 2009). Wsp and ISNew PCR do not produce amplicons in all samples except for the positive control (A, B). Tubulin PCR, however, shows bright signals in all samples (C). Positive controls for PCR are Wolbachia high titer G. morsitans centralis denoted with (+), not that both gene targets were detected with this DNA template. The (M) designates the 1 kb DNA ladder used as size reference. (DOCX) [file pone.0061150.s004.docx]
